# Effects of Servo Tensile Test Parameters on Mechanical Properties of Medium-Mn Steel

**DOI:** 10.3390/ma12223793

**Published:** 2019-11-19

**Authors:** Xuemin Chi, Shuo Han

**Affiliations:** State Key Laboratory of Structural Analysis for Industrial Equipment, School of Automotive Engineering, Dalian University of Technology, Dalian 116024, China; hanshuo@mail.dlut.edu.cn

**Keywords:** total elongation, medium-Mn steel, servo tensile parameters, artificial neural networks

## Abstract

As a new type of third-generation automotive steel with high strength and plasticity, medium-Mn steel (MMnS) has been widely used in automotive industries for its excellent properties. In recent years, servo stamping technology for high-strength metal forming is a hot topic due to its good performance in forming under complex processing conditions, and servo parameters determine the forming quality. In this paper, experiments considering tensile speed and position where speed changes (PSC) were carried out on MMnS to investigate the influences of tensile parameters on mechanical properties including strength and total elongation (TE). The results show that PSC does not significantly impact total elongation. Initial tensile speed (ITS) and final tensile speed (FTS) significantly impact the total elongation. The interaction between all tensile parameters can impact total elongation. Two artificial neural networks, back propagation neural network (BPNN) and radial basis function neural network (RBFNN), were used to establish analytical models. The results of supplemental experiment and residual analysis were conducted to verify the accuracy of the analytical models. The BPNN has a better performance and the analytical model shows that with the increase of PSC, it has a slight impact on the changes of optimal and minimum total elongation, but the combinations of tensile parameters to obtain total elongations higher than 40% change significantly.

## 1. Introduction

**Figure 1:**
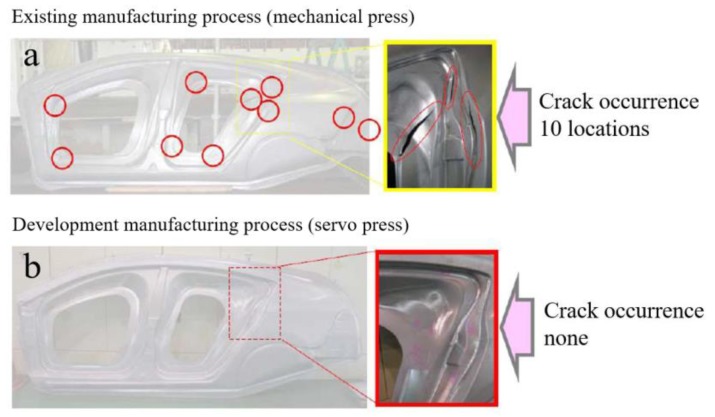
Stamping results of an automotive side board. (**a**) The result of mechanical press; (**b**) The result of servo press.

Stamping forming of sheet metal is a very significant process for the manufacture of automotive parts. The sheet metal is formed into the shape of the mold under the pressure of the slide [1,2,3]. The traditional stamping process is direct stamping; the slide of the mechanical press is directly stamped to the mold due to the simple slide motion system. For some complex shape automotive parts, multi-stage forming has to be performed [4]. However, there are many disadvantages in mechanical stamping and multi-stage processes. The stress–strain distribution on the sample is not uniform, and the stress concentration at some locations caused by direct stamping to the mold results in cracking [5]. The multi-stage process can release the residual restress to a certain extent, which improves the forming quality, but it has poor flexibility and the investment of mold, equipment, and time costs is enormous. Servo stamping forming is a new forming technology using a servo press [6,7]; the user-defined slide motion or other different motion modes can guarantee the sheet metal is optimally loaded in the stamping process, which solves the problems existing in mechanical stamping. For example, the automotive side board stamped by mechanical stamping has many cracks, shown in Figure 1a. However, the development of servo stamping forming provides optimized loading conditions for stamping, which shows a good performance, shown in Figure 1b.

Many researches were focused on servo stamping forming, the V-shaped and U-shaped bending [8,9,10], and deep drawing [11,12,13]. In the process of servo stamping forming, involved parameters are slide velocity, slide holding time and force, and slide displacement. Among these servo parameters, the slide speed and the position where speed changes (PSC) play a crucial role in the stamping forming quality. Hayashi et al. [14] investigated the effect of forming speed on formability of 6000 series Al–Mg–Si alloy. Their research showed that the formability of aluminum alloy was improved with the increase in forming rate, and the relation between slide speed and PSC indicated an acceleration effect existed on the formability. Similar research has been carried out on Al 5182-O by Ju et al. [15]; the results showed that better part quality could be achieved with a higher forming speed, which could be considered as a more efficient process in production. Mori et al. [16] performed cold stamping and hot stamping with different stamping speed on ultra-high-strength steel. The results showed that the application of servo forming to hot stamping was attractive to improve the formability due to the high speed. However, in the above research, the setting of the servo stamping parameters basically relied on the experience level of the technicians, and the determination of related parameters lacked systematic theoretical guidance and consequently had to be obtained through continuous adjustment.

Medium-Mn steel (MMnS) is a third-generation automotive steel with excellent strength and plasticity. With the increasing requirement for lightweight and safety in the automotive industry, MMnS has become a promising steel in the manufacturing industry [17,18]. However, the researches were mainly focused on micro-structure and on mechanical properties in tensile tests involved no speed combinations [19,20,21]. The properties of MMnS under the complex servo parameters mechanism have not been reported yet. In order to guide the application of MMnS in servo stamping, it is significant to study the effects of servo parameters on mechanical properties.

In this paper, the servo tensile test is used to study mechanical properties of MMnS under different initial tensile speed (ITS), final tensile speed (FTS) and PSC parameters. Furthermore, artificial neural networks and experiments are combined to analyze the influencing rule of servo parameters on mechanical properties and achieve the optimal tensile parameters.

## 2. Experimentation

### 2.1. Materials

In this paper, the MMnS was developed by Central Iron and Steel Research Institute (CISRI) of China. Its chemical composition is listed in Table 1. The thickness of tested steels was 1.6 mm.

**Figure 2:**
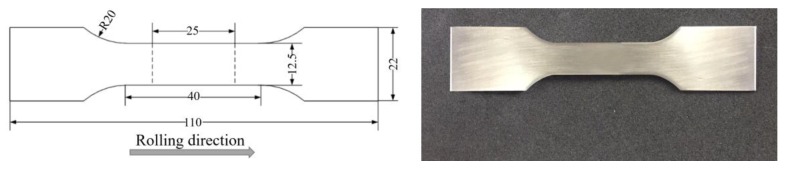
Sample dimension for uniaxial tensile test and post-fracture specimen.

The uniaxial tensile tests were conducted for the MMnS in a universal testing machine. Its maximum testing speed was 500 m/min. The extensometer was used to measure the material deformation. The gauge length was 25 mm and the strain value was acquired through conversion of the corresponding deformation. The tensile samples were designed in accordance with the international standard [22]. The sample dimension and post-fracture specimen are shown in Figure 2. The tensile specimen was manufactured along the rolling direction in order to keep the same anisotropy for all the tests.

### 2.2. Determination of Process Parameters

The ranges of servo tensile test parameters of speed and position where speed changes are determined in this section. To investigate the influence of important parameter speed and PSC on mechanical properties of MMnS, the speed was designed by the following considerations: First, the range of tensile speed has to be large due to the high actual stamping speed in the processing of automotive parts. Second, the tensile speed was selected within the range of the servo press. Therefore, tensile velocities were selected to be 3 mm/min, 15 mm/min, 75 mm/min, 150 mm/min, and 300 mm/min, and all tests were repeated three times. The MMnS properties such as tensile strength, yield strength, and total elongation were obtained from the stress–strain curves. 

**Figure 3:**
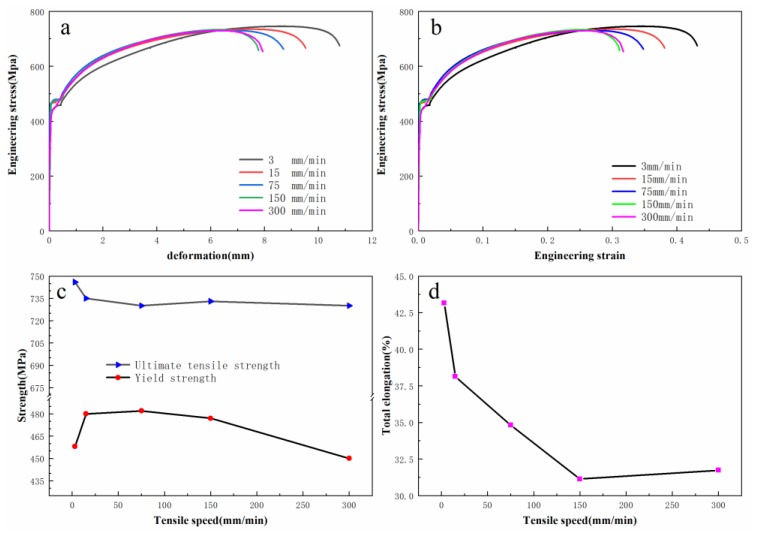
Results of the single-speed test. (**a**) Stress-deformation curve; (**b**) Engineering stress-strain curve; (**c**) Changes of strength; (**d**) Changes of total elongation.

The test results are shown in Figure 3. The yield strength and ultimate tensile strength of MMnS were less influenced by the tensile speed, and the total elongation was greatly influenced. With the increase of tensile speed, the total elongation tended to decrease. It could be seen that the total elongation tended to be consistent. Considering that the larger total elongation is beneficial to the formability in actual forming process, 3 mm/min, 15 m/min, and 75 mm/min were selected as the speed parameters in this paper. 

The parameter of PSC is another important parameter. Its selection was based on two conditions. First, its range had to be as large as possible to facilitate the observation of the influence on total elongation. Second, after speed changing, FTS had to be executed with a certain amount of deformation. In Figure 3, it is shown that necking occurred when the deformation reached 7 mm. The successful sheet metal forming required the deformation to be in the uniformly-deformed region before the occurrence of necking, so as to avoid excessive thinning of automotive parts and failure of the materials. Therefore, the maximum PSC value was set as 6 mm. Three position values of 3 mm, 4.5 mm, and 6 mm were designed as experimental parameters. 

### 2.3. Servo Tensile Test Setup

**Figure 4:**
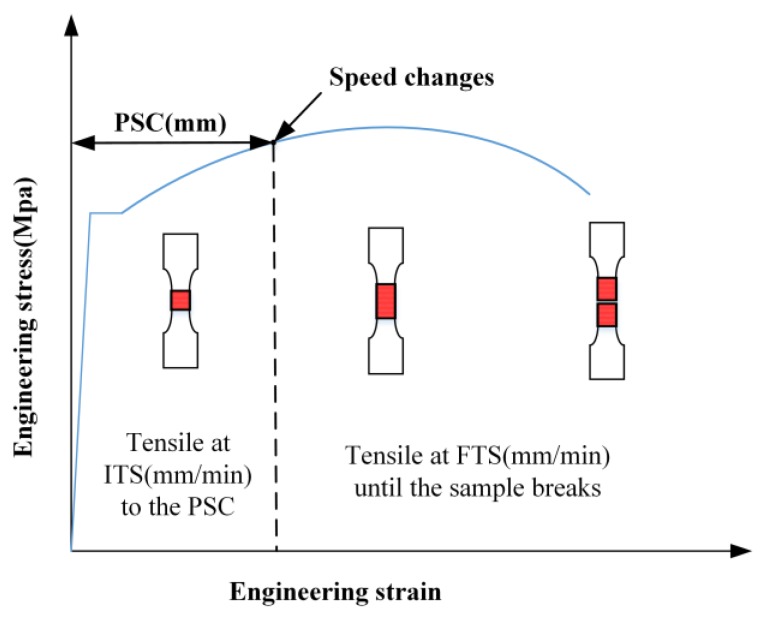
Schematic diagram of servo tensile.

The range of three parameters, including initial tensile speed, final tensile speed, and position where speed changes (PSC) were designed based on the above investigation. The schematic diagram of the servo tensile is shown in Figure 4. Eighteen combinations of experimental conditions were designed and the detailed test conditions are shown in Table 2. In this paper, each experimental condition was performed three times, therefore, 54 experiments in total were conducted to observe the variation of total elongation. 

## 3. Effect of Process Parameters on Elongation

### 3.1. Servo Tensile Results and Data Analysis

**Figure 5:**
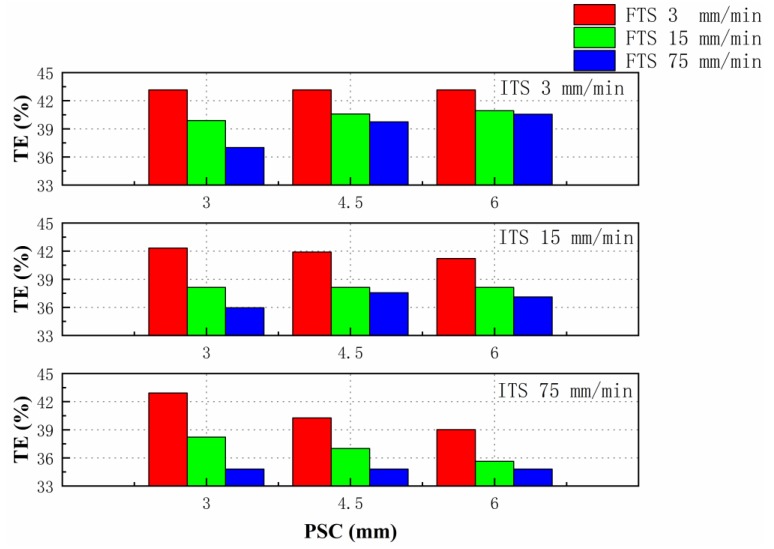
Histogram plots of servo tensile tests and single-speed tests.

The results of servo tensile tests are shown in the histogram of Figure 5. In order to further analyze results, there are extra 3 columns of histograms from the single-speed test results in each histogram, so a total of 9 more columns were added for comparison.

According to Figure 5, the increase of FTS caused the reduction of total elongation under any circumstances. From the analysis of vertical direction (ITS 3, 15, and 75 mm/min), the influence of ITS on total elongation was dependent on the values of PSC and FTS. From the histogram of the same color, the influence of PSC on the total elongation can be observed, and the cases when ITS and FTS were equal are not considered. The total elongation increased with the increase of PSC when ITS was 3 mm/min (green with the deltas of 0.7% and 0.36% and blue with the deltas of 2.74% and 0.81%). When ITS was 15 mm/min, the total elongation decreased when FTS is 3 mm/min (red with the deltas of −0.42% and −0.70%). It shows a rise and fall trend when FTS was 75 mm/min (blue with the deltas of 1.6% and −0.45%). When ITS was 75 mm/min, the total elongation maintained a consistent downward trend (red with deltas of −2.65% and −1.25% and green with deltas of −1.21% and −1.38%). Therefore, there were three kinds of rules on the tensile total elongation with the increase of PSC.

The overall pattern of tensile parameters influencing the total elongation of MMnS was observed. To further analyze the influences of servo tensile parameters and their interactions on the total elongation, analysis of variance (ANOVA) was carried out in the following section. 

### 3.2. Analysis of Variance

ANOVA is a classic method used in experimental and statistical data analysis [23]. It is mainly taken to explore which factors have a significant impact on the indicators. Here, three analyses including main effects, interaction plots, as well as ANOVA, are performed for further understanding the influence of process parameters on the mechanical properties of MMnS.

**Figure 6:**
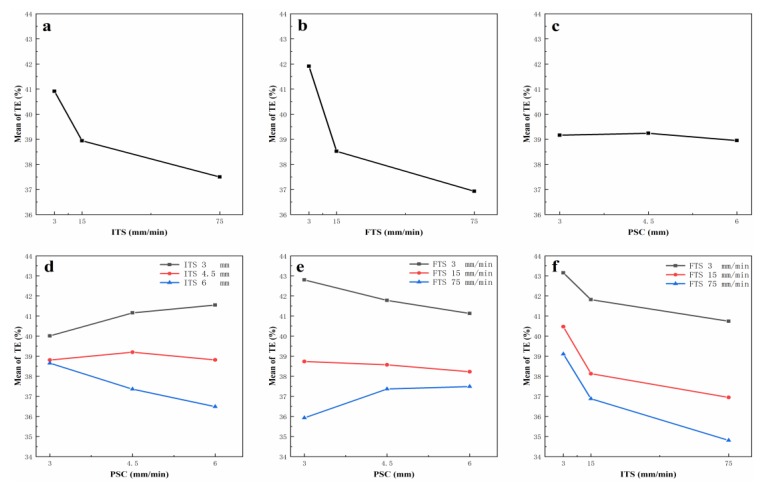
Main effects and interaction plots for total elongation. (**a**) The main effect of ITS; (**b**) The main effect of FTS; (**c**) The main effect of PSC; (**d**) The interaction effect between ITS and PSC; (**e**) The interaction effect between FTS and PSC; (**f**) The interaction effect between FTS and ITS.

As shown in the main effects plot in Figure 6a–c, the variation range of total elongation caused by FTS was larger than those caused by other two parameters. With the increase of ITS or FTS, the variation of mean total elongation both showed downtrend. PSC had minor influence on elongation. This result is also confirmed by the rule in servo tensile experiments in Figure 5. According to the interaction plots in Figure 6d–f, the interaction between ITS and PSC and FTS and PSC showed uptrend or downtrend, which depended on the value of the slide speed.

The ANOVA analysis results of tensile parameters is shown in Table 3, where df is the degrees of freedom of each factor, Standard deviation square (SS) is the sum of squares of differences between factor level means and overall mean. Mean square (MS) is defined as SS divided by df, and F is defined as MS divided by error of MS. The desired level of confidence was considered to be 95%. Values of F larger than critical value of F indicates that the correspondent term is significant. The critical value of $F_{0.95;2,8}$ is 4.46 and $F_{0.95;4,8}$ is 3.84. In this case, from data of main effects, FTS had the most prominent effect on the elongation, followed by ITS. The PSC had the least significant effect on the elongation. As for the interaction effect, the interaction F value of ITS and position where speed changes was 29.78, which is slightly greater than the F value of finial tensile speed and position where speed changes, 24.27. The interaction of ITS and FTS also impacted the elongation, but not as significantly as other combinations. This result is also confirmed by the main and interaction plots shown in Figure 6a–f.

## 4. Artificial Neural Network

In this section, two different artificial neural networks were adopted to obtain the calculation model of total elongation (TE) based on servo tensile parameters and investigate the influences of tensile parameters on total elongation. The training data of the neural network consisted of 27 groups, 18 of which were from the servo tensile tests, and the other 9 were from single-speed tensile results, which had the same ITS and FTS at different PSC. The purpose of extra training data were to train the neural network to obtain an ability to recognize that the elongations were equal when ITS equals FTS. The training data are shown in Table 4.

### 4.1. Calculation Model of Total Elongation Based on Servo Tensile Parameters

**Figure 7:**
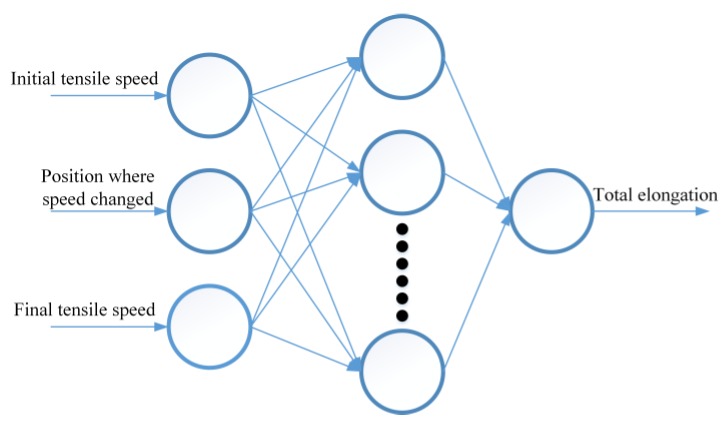
Artificial neural network (ANN) structure of total elongation prediction.

Artificial neural network (ANN) is a distributed algorithm based on neural network of biology [24]. The advantage of ANN not requiring any explicit formula or calculation model makes itself a very effective method for this paper. Many scholars have obtained the optimal parameters in different forming fields by neural networks [25,26,27,28]. Research into mechanical properties of MMnS under servo conditions is at an initial stage and the mechanism is not quite clear. The applications of numerical simulation and mathematical modeling are limited. In this study, back propagation neural network (BPNN) and radial basis function neural network (RBFNN) were adopted to predict and analyze the changes of total elongation in servo tensile tests. The ANN structure of total elongation prediction for MMnS is shown in Figure 7.

#### 4.1.1. BPNN and RBFNN Modelling 

Back propagation is a multi-layer feedforward neural network, which is characterized by transferring the signal forward and propagating the error backward. The calculation process is as follows:

(1) $${ℋ}_{j}=f(\sum_{i=1}^{n}w_{ij}x_{i}-a_{j})\;\;j=1,2,\ldots ,J$$

where ${ℋ}_{j}$ denotes jth output of neurons in the hidden layer, f(⋅) is the sigmoid transition function, $w_{ij}$ denotes the weights between the input and hidden layer, $a_{j}$ denotes the threshold between the input layer and hidden layer. n is the number of neurons in the input layer, and J is the number of neurons in the hidden layer.

(2) $$O_{k}=\Phi (\sum_{j=1}^{J}w_{jk}{ℋ}_{j}-b_{k})\;\;k=1,2,\ldots ,K$$

where $O_{k}$ denotes the jth output of neurons in the output layer, Φ(⋅) denotes the purelin transition function in the output layer, $w_{jk}$ is the weights between the hidden layer and output layer, $b_{k}$ denotes the threshold of the output layer, and K is the number of neurons in the output layer.

(3) $$E=\frac{1}{2}\sum_{k=1}^{K}{\left(r_{k}-O_{k}\right)}^{2}$$

(4) $$\Delta w_{ij}=\eta {ℋ}_{j}\left(1-{ℋ}_{j}\right)x_{i}\sum_{k=1}^{K}w_{jk}(r_{k}-O_{k})\;\;i=1,2,\ldots ,n\;\;j=1,2,\ldots ,J$$

(5) $$\Delta a_{j}=\eta {ℋ}_{j}\left(1-{ℋ}_{j}\right)\sum_{k=1}^{K}w_{jk}(r_{k}-O_{k})\;\;j=1,2,\ldots ,J$$

(6) $$\Delta b_{k}=r_{k}-O_{k}\;\;k=1,2,\ldots ,K$$

where E denotes the total error of the network, $r_{k}$ is desired output, and η is the learning rate. 

RBFNN [29] can model any nonlinear function using a single hidden layer, which eliminates considerations of determining the number of hidden layer and neurons [30]. The operation of the output layer was as follows:

(7) $$Y_{ik}=b_{k}+\sum_{j=1}^{J}w_{jk}\varphi (x,x_{j})\;\;i=1,2,\ldots ,n\;\;k=1,2,\ldots ,K$$

where n denotes the number of input samples, J denotes the number of neurons in the hidden layer. K denotes the number of neuron in the output layer. *b_k_* is bias, $w_{jk}$ is the weight between the hidden layer and the output layer. $\varphi (x,x_{j})$ is the radial basis function, and $x_{j}$ is the jth center of hidden neurons. The radial basis function used in this paper is given below:

(8) $$\varphi (x,x_{j})=\exp\left(-\frac{\left\|x-x_{j}\right\|}{2\sigma_{j}^{2}}\right)\;\;j=1,2,\ldots ,J$$

where ‖·‖ is the Euclidean norm, and $\sigma_{j}$ is the width of jth neurons in hidden layer.

#### 4.1.2. Neural Network Setup

**Figure 8:**
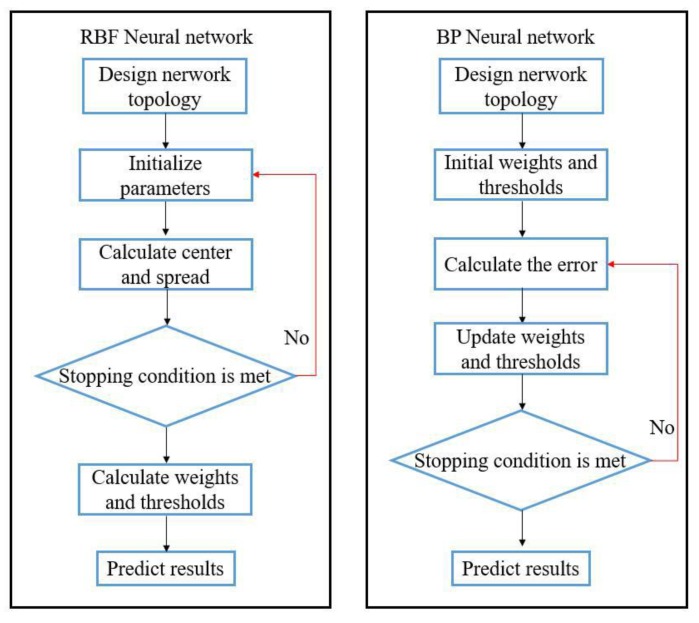
Flow chart of RBFNN and BPNN.

The flowchart of RBFNN and BPNN is shown as Figure 8.

Training the RBFNN consisted of two stages. In the first stage, an unsupervised, clustering algorithm called the k-means algorithm was used. In the second stage, the width of radial basis function (also called spread) was frequently optimized and the weights between the hidden and output layers were both determined in a heuristic way. The learning object was 0.01, and the spread was iterated from 1. BPNN is a universal network, which can approximate the objective function with desired precision in theory [31]. However, it is found that most of the time, it is difficult to obtain a satisfactory network by manually adjusting parameters. The cyclic repetition training by setting the accuracy requirements of the network is recommended. The number of iterations was set to 100, with a learning rate of 1% and a learning objective of 0.01. The Levenberg-Marquardt (LM) optimization algorithm [32] was applied for network training. Data normalization was carried out to eliminate the difference caused by the data magnitude in each dimension. The results and plots of the distribution of elongations were obtained in data processing and simulation running Matlab (2018a, MathWorks, Natick, MA, USA).

### 4.2. Prediction of Total Elongation by Neural Networks

#### 4.2.1. Comparison of Prediction Results

**Figure 9:**
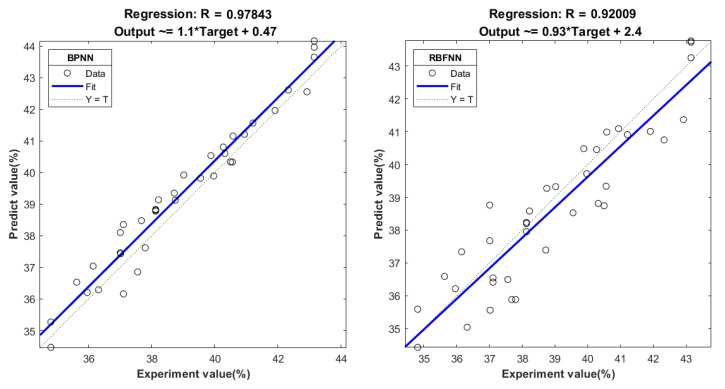
Regression analysis between experimental values and predicted values.

To verify the accuracy of network training, another 10 groups of parameters were randomly selected for the servo tensile test, and predicted values of two networks were compared. The tensile parameters and comparison results are shown in Table 5. The analysis of regression between experimental values (include the training data) and predicted values is shown in Figure 9.

**Figure 10:**
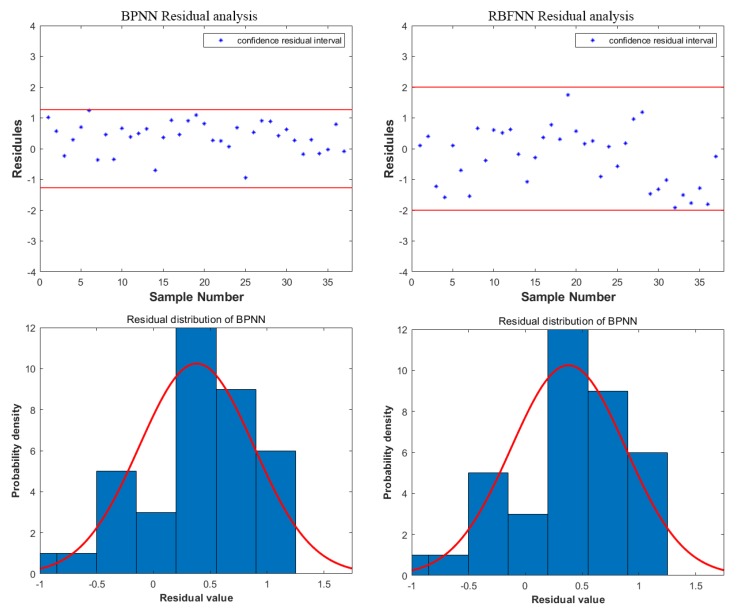
The residual analysis result and the probability density of the residual.

According to Table 5, the predicted error of BPNN and RBFNN was less than 1% and 2%, respectively. Both of the prediction trends showed a good agreement with the experiment. The overall error of BPNN was less than RBFNN. In Figure 9, the correlation coefficients R of BPNN and RBFNN between experimental values and predicted values were 0.978 and 0.920, respectively. The results of regression analysis show that the correlation between the observed values and the corresponding predicted values was significant. To further verify the accuracy of the prediction models, a residual analysis was performed. The residual was defined as:

(9) $$e_{i}=y_{i}-{\tilde{y}}_{i}$$

where $y_{i}$ is experimental total elongation and ${\tilde{y}}_{i}$ is the predicted total elongation. The confidential residual interval was defined as:

(10) $$-2\hat{\sigma }<e_{i}<2\hat{\sigma }$$

where $\hat{\sigma }$ is the standard deviation of the residual. It was defined as:

(11) $$\hat{\sigma }=\sqrt{\frac{\sum_{i=1}^{n}e_{i}^{2}}{n-1}}$$

where n is the number of samples (in this paper n = 37). The prediction model proved accurate only when all of $e_{i}$ were in the confidential residual interval. Otherwise the model needed adjustment to reach the requirement. The residual analysis results of BPNN and RBFNN are shown in Figure 10.

For BPNN, the sample mean and standard deviation of the residuals were 0.38 and 0.63, respectively. The confidential residual interval was between −1.26 and 1.26. For RBFNN, the sample mean and standard deviation of residuals were 0.82 and 1.01, respectively. The confidential residual interval was between −2.02 and 2.02. According to above results, all of $e_{i}$ were in confidential residual intervals. The probability density of the residual was accordance with normal distribution. Both BPNN and RBFNN were reliable and accurate in predicting the total elongation. However, the error of BPNN was smaller, the interval was narrower and the correlation coefficient R was higher. Therefore, the performance of BPNN was better. 

#### 4.2.2. Result Analysis

**Figure 11:**
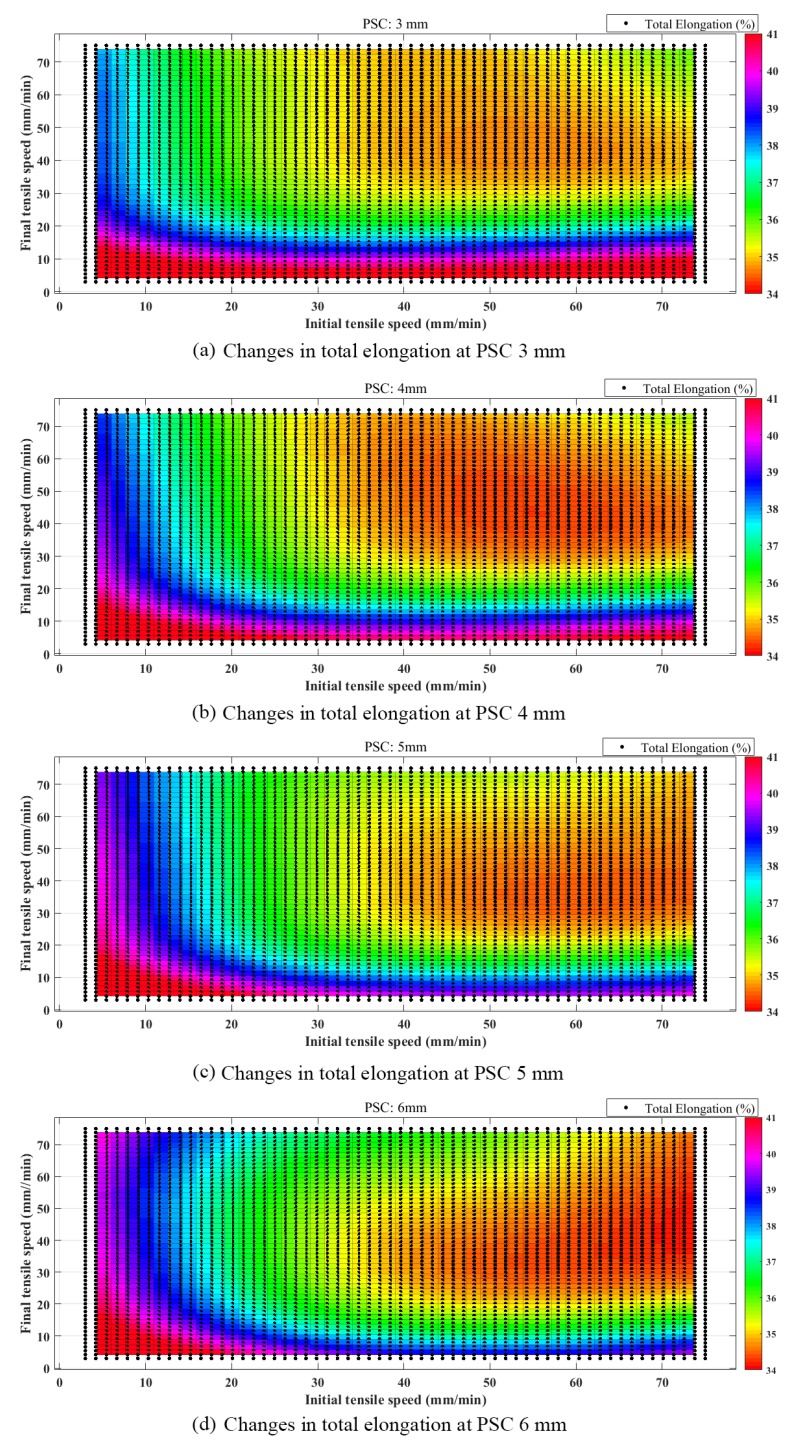
The distribution of total elongation at different PSC.

When the PSC is 3, 4, 5, and 6 mm, the distributions of the total elongation along with the ITS and FTS are shown in Figure 11a–d. 

Colors are used to represent the gradually increasing total elongation in the order of orange, green, blue, and red. Among them, the dark red area is the optimal area of tensile strength. The optimal ranges of ITS and FTS for different PSC are presented in Table 6.

1. For 3 mm PSC, the optimal range of ITS was 3–75 mm/min and FTS was 3–17 mm/min. The total elongation could reach 41% in the optimal range. The maximum total elongation was 44.64%. Low total elongation was concentrated at ITS of 25–75 mm/min and FTS of 30–75 mm/min. The minimum total elongation was 34.2%.
2. For 4 mm PSC, the optimal range of ITS was 3–75 mm/min and FTS was 3–25 mm/min. The total elongation could reach 40% in the optimal range. The maximum total elongation was 44.05%. Low total elongation was concentrated at ITS of 25–75 mm/min and FTS of 25–75 mm/min. The minimum total elongation was 34.2%.
3. For 5 mm PSC, the optimal range of ITS was 3–37 mm/min and FTS was 3–32 mm/min. The total elongation could reach 40% in the optimal range. The maximum total elongation was 44.36%. Low total elongation was concentrated at ITS of 30–75 mm/min and FTS of 20–75 mm/min. The minimum total elongation was 34.2%.
4. For 6 mm PSC, the optimal range of ITS was 3–20 mm/min and FTS was 3–25 mm/min. The total elongation could reach 40% in the optimal range. The maximum total elongation was 44.36%. Low total elongation was concentrated at ITS of 35–75 mm/min and FTS of 18–75 mm/min. The minimum total elongation was 34.2%.

According to analysis of the predicted results of servo tensile by the BPNN, the influences of servo tensile parameters on the total elongation are summarized as below.

- With the increase of PSC, the changes of maximum and minimum total elongations were limited, but the combinations of servo tensile parameters to obtain a total elongation higher than 40% changed significantly.
- The variation trend of optimal servo tensile parameters was: with the increase of PSC, the optimal tensile parameters from higher ITS and lower FTS shifted to lower ITS and higher FTS gradually.
- For 3 and 4 mm PSC, the influence of ITS on total elongation was not significant.

## 5. Conclusions

In servo tensile tests of MMnS, the influences of tensile parameters on total elongation are complicated due to the complex servo tensile mechanism. The analysis of main effect plots, interaction plots, and variance were adopted to study the weight of each servo tensile parameter and their interaction. The prediction models of BPNN and RBFNN were verified and compared. The analytical model of BPNN with better performance was used to analyze the detailed impacts of servo tensile parameters. The following conclusions are presented.

The influence of servo tensile parameters on the mechanical properties of MMnS was mainly reflected in the total elongation. The impact on yield strength and ultimate tensile strength was not significant and can be neglected. By controlling the servo tensile parameters, with the premise of ensuring high strength of MMnS, high plasticity and forming performance could be obtained.

Three kinds of rules governing the total elongation of MMnS were obtained according to the value of ITS. The first rule: ITS is small, and as the PSC increases, the total elongation increases. The second rule: ITS is around 15 mm/min. As the PSC increases, the total elongation may increase (FTS = 3 mm/min) or decrease (FTS = 75 mm/min). The third rule: ITS is around 75 mm/min, and as the PSC increases, the total elongation decreases.

BPNN with a better performance was used to further analyze the influences of servo tensile parameters considering total elongation and stamping time comprehensively. To obtain elongations higher than 40%, with the increase of PSC, the optimal tensile parameters from any ITS and lower FTS shifted to lower ITS and higher FTS. The variation of PSC had little impact on the maximum and minimum total elongation, but significant influence on the combinations of ITS and FTS to obtain a higher total elongation. 

## Figures and Tables

**Table 1:** Chemical composition of the tested material (wt.%).

C	Mn	AL	S	P	Fe
0.1	5.0	0.03	0.02	0.008	Balance

**Table 2:** The combination of different servo tensile parameters.

Test	Initial Tensile Speed mm/min	Position where Speed Changes mm	Final Tensile Speed mm/min
1	3	3	15
2	3	3	75
3	15	3	3
4	15	3	75
5	75	3	3
6	75	3	15
7	3	4.5	15
8	3	4.5	75
9	15	4.5	3
10	15	4.5	75
11	75	4.5	3
12	75	4.5	15
13	3	6	15
14	3	6	75
15	15	6	3
16	15	6	75
17	75	6	3
18	75	6	15

**Table 3:** ANOVA analysis results.

Source	df	SS	MS	F
ITS	2	52.673	26.336	289.4
FTS	2	116.008	58.004	637.4
PSC	2	0.400	0.200	2.2
ITS*FTS	4	2.989	0.747	8.2
ITS*PSC	4	10.834	2.708	29.78
FTS*PSC	4	8.836	2.209	24.27
Error	8	0.726	0.091	–
Total	26	192.465	–	–

**Table 4:** Data for neural network training.

Test	ITS (mm/min)	PSC (mm)	FTS (mm/min)	TE (%)
1	3	3	3	43.15
2	3	3	15	39.88
3	3	3	75	37.01
4	3	4.5	3	43.15
5	3	4.5	15	40.58
6	3	4.5	75	39.75
7	3	6	3	43.15
8	3	6	15	40.94
9	3	6	75	40.56
10	15	3	3	42.33
11	15	3	15	38.13
12	15	3	75	35.96
13	15	4.5	3	41.91
14	15	4.5	15	38.13
15	15	4.5	75	37.56
16	15	6	3	41.21
17	15	6	15	38.13
18	15	6	75	37.11
19	75	3	3	42.92
20	75	3	15	38.22
21	75	3	75	34.81
22	75	4.5	3	40.27
23	75	4.5	15	37.01
24	75	4.5	75	34.81
25	75	6	3	39.02
26	75	6	15	35.63
27	75	6	75	34.81

**Table 5:** Results and comparison.

Test	ITS (mm/min)	PSC (mm)	FTS (mm/min)	Test Value of TE %	Predicted Value of BPNN %	Predicted Value of RBFNN %	Error	
							BP	RBF
1	10	3.5	75	36.15	37.04	37.34	0.89	1.19
2	10	3.5	50	37.02	37.43	35.56	0.41	1.46
3	3	4	50	38.72	39.35	37.39	0.63	1.33
4	3	4	30	39.55	39.82	38.53	0.27	1.02
5	15	4.5	30	37.80	37.62	35.88	0.18	1.91
6	30	4.5	3	40.32	40.61	38.81	0.29	1.51
7	30	5.0	3	40.50	40.34	38.74	0.16	1.76
8	50	5.0	15	36.32	36.29	35.04	0.03	1.28
9	10	5.5	50	37.68	38.48	35.88	0.8	1.80
10	75	5.5	3	39.97	39.89	39.72	0.08	0.25

**Table 6:** Optimal and worst range for different position where speed changes (PSC).

PSC (mm)	Optimal Range for Total Elongation			Worst Range for Total Elongation		
	ITS (mm/min)	FTS (mm/min)	Maximum (%)	ITS (mm/min)	FTS (mm/min)	Minimum (%)
3	3–75	3–17	44.64	25–75	30–75	34.20
4	3–75	3–19	44.05	25–75	25–75	33.89
5	3–25	3–20	44.36	30–75	20–75	33.98
6	3–20	3–25	44.13	35–75	18–75	33.55
